# The type 1 submovement conundrum: an investigation into the function of velocity zero-crossings within two-component aiming movements

**DOI:** 10.1007/s00221-024-06784-0

**Published:** 2024-02-08

**Authors:** James W. Roberts, James J. Burkitt, Digby Elliott

**Affiliations:** 1 Brain and Behaviour Research Group, Research Institute of Sport and Exercise Sciences (RISES), Liverpool John Moores University, Tom Reilly Building, Byrom Street, Liverpool, L3 5AF UK; 2 School of Health Sciences, Psychology, Action and Learning of Movement (PALM) Laboratory, Liverpool Hope University, Hope Park, Liverpool, L16 9JD UK; 3 Department of Kinesiology, McMaster University, 1280 Main Street West, Hamilton, ON L8S 4K1 Canada

**Keywords:** Speed-accuracy, Overshoot, Correction, Feedback, Feedforward

## Abstract

In rapid manual aiming, traditional wisdom would have it that two components manifest from feedback-based processes, where error accumulated within the primary submovement can be corrected within the secondary submovement courtesy of online sensory feedback. In some aiming contexts, there are more type 1 submovements (overshooting) compared to types 2 and 3 submovements (undershooting), particularly for more rapid movements. These particular submovements have also been attributed to a mechanical artefact involving movement termination and stabilisation. Hence, the goal of our study was to more closely examine the function of type 1 submovements by revisiting some of our previous datasets. We categorised these submovements according to whether the secondary submovement moved the limb closer (functional), or not (non-functional), to the target. Overall, there were both functional and non-functional submovements with a significantly higher proportion for the former. The displacement at the primary and secondary submovements, and negative velocity peak were significantly greater in the functional compared to non-functional. The influence of submovement type on other movement characteristics, including movement time, was somewhat less clear. These findings indicate that the majority of type 1 submovements are related to intended feedforward- and/or feedback-based processes, although there are a portion that can be attributed an indirect manifestation of a mechanical artefact. As a result, we suggest that submovements should be further categorised by their error-reducing function.

## Introduction

It has long been known that stereotypical discrete target-directed manual aiming movements are comprised of two components: initial impulse and current control (Woodworth 1899). The initial impulse is marked by a ballistic reach that represents the distance-covering portion of the movement; also referred to as the primary submovement. Current control features a slower and perhaps more iterative profile that represents a correction or corrections to the movement courtesy of online sensory feedback; also referred to as the secondary submovement (see also Crossman and Goodeve 1983; Elliott et al. 2001; Keele 1968; Meyer et al. 1988). It is these precise submovement features that underpin the speed-accuracy trade-off, where there is an inverse relation between the movement speed/impulse magnitude and capacity to hit the target/movement precision (Fitts 1954; Fitts and Peterson 1964; Schmidt et al. 1979). Along these lines, it has been shown that a higher proportion of submovements manifest when aiming to small compared to large targets (Dounskaia et al. 2005; Fradet et al. 2008a, b; Roberts 2020), and with vision compared to no vision (Hsieh et al. 2022; Khan and Franks 2003; Woodworth 1899; cf. Elliott et al. 1991). Of interest, the different types of submovement that can take place are threefold: reversals, second accelerations and discontinuities/braking. These submovements are marked by zero-crossings during the deceleration phase in velocity, acceleration and jerk, respectively. Hence, as first, second and third derivates of displacement, they can be referred to as type 1, type 2 and type 3 submovements, respectively.

With this in mind, subsequent models have attempted to elucidate the manifestation of submovements. Most notably, there is the optimised submovement model, whereby the primary (sub) movement tends to land near target-centre so as to maximise the chances of hitting the target, whilst any movements landing under or over the target can be corrected by a secondary submovement (Meyer et al. 1988; see also, Slifkin and Eder 2017). In this regard, there is an equal distribution of submovements that come from initially undershooting (type 2, type 3) and overshooting (type 1) the target. Alternatively, there is the minimisation model, which states that the primary submovement tends to undershoot the target so as to not completely overturn inertia, and limit the time and energy-expenditure of the subsequent correction with the secondary submovement (type 2, type 3 > type 1) (Elliott et al. 2004; see also the multiple process model, Elliott et al. 2010, 2017; for a theoretical argument attempting to reconcile these two points of view, see Roberts et al. 2022).

Though it is clear that primary submovements often undershoot the target to avoid the time and energy-expenditure associated with a potential target overshoot (e.g. Bennett et al. 2012; Burkitt et al. 2015, 2017; Elliott et al. 2004; Engelbrecht et al. 2003; Lyons et al. 2006), there are also situations where a large proportion of aiming movements include type 1 submovements. For example, we have shown a greater incidence of overshooting and subsequent type 1 submovements when targets are sized according to one’s own inherent variability (Roberts et al., 2021), under high-performance-related stress (Roberts et al. 2018) and aiming to the last-placed target in an array of placeholders (Roberts et al. 2016). At the same time, these effects were attributed to the possibility of accumulating more potential energy with a view to taking advantage of the viscoelastic properties at the opposing antagonist muscle. This logic is primarily adapted from findings within continuous reciprocal aiming, where there is the possibility to make faster and smoother transitions as opposed to initiating excess decelerative forces with a view to terminating the moving limb (Adam et al. 1993; Guiard 1993; Savelberg et al. 2002). It is these precise same processes that may be responsible for modifying the limb position following an overshoot within the alternative discrete aiming context that is of interest to the present study.

However, it has been suggested elsewhere that at least some type 1 submovements are the result of a non-functional mechanical artefact (Dounskaia et al. 2005; Fradet et al. 2008a; see also Plamondon and Alimi 1997; cf. Elliott et al. 2017; Keele 1968; Meyer et al. 1988; Woodworth 1899). Specifically, the type 1 submovement may indirectly manifest as a by-product of a movement termination process, where mechanical oscillations unfold as a result of trying to “clamp” the limb near the target. If so, then it is reasonable to assume that differences in the type of submovements will begin to emerge when altering the underlying aiming task dynamics with a view to decoupling movement accuracy and termination. For example, by retaining the need for accuracy, but without limb acceleration having to reach zero such as within a reciprocal aiming task, where the limb is reversed at the target to move back towards the home position (assuming the movements remain fully harmonious or cyclical; Buchanan et al. 2006; Guiard 1993), we find a decrease in the incidence of type 1 submovements (Fradet et al. 2008a; see also Dounskaia et al. 2005). In addition, by limiting the need for accuracy, but retaining the termination of the moving limb such as within a passing task, where the limb runs through the target before stopping in open space (for an alternative view on the control processes underpinning “passing”, see Khan and Binsted 2010), we find an increase in the incidence of type 1 submovements.

In line with this suggestion, it was also found that a higher proportion of type 1 submovements took place when aiming to large compared to small targets, where the movements happened to be more rapid (Dounskaia et al. 2005; Fradet et al. 2008a; Hsieh et al. 2017). This trend appeared to be even more robust for temporally compared to accuracy-constrained aiming movements that involved an even larger range of movement times (approx. 420–2115 ms; Hsieh et al. 2019). Likewise, the higher proportion of type 1 submovements found for young healthy adults compared to the elderly (Fradet et al. 2008b) and Parkinson’s patients (Dounskaia et al. 2009) may be attributed to their much shorter movement times. Meanwhile, despite finding fewer type 1 submovements when aiming with vision compared to no vision (Hsieh et al. 2022), further inspection highlighted how this trend was primarily contaminated by variations in movement time as there was an incidentally shorter movement time for the latter. As a result, it seems the previously identified “clamping” at movement termination is even further evident when it follows faster movements due to the need for a greater counter-acting force to dampen the higher magnitude velocity.

Taken together, it is of interest to further examine the datasets from our previously mentioned studies; that is, when type 1 submovements tended to dominate (Roberts et al. 2016, 2018, 2022). Specifically, to further examine the function of type 1 submovements, we isolated the incidences of type 1 submovements, and further categorised them as functional and non-functional based on whether the secondary submovement moved the limb closer to the target than the primary submovement (see Khan et al. 2006; Robinson et al. 2014). Although there may be a confluence of both functional and non-functional submovements (for a similar suggestion, see Dounskaia et al. 2005), it is possible that the submovements in question could be more greatly associated with one category over another. Namely, if there are substantially more functional compared to non-functional type 1 submovements, then it would mostly advocate for these submovements being a direct manifestation of feedback- or feedforward-based modification to reach closer to the target. On the other hand, the opposite trend would suggest a mostly mechanical explanation, where submovements are a mere by-product of some biomechanical constraint. In addition, we analysed other outcome and kinematic measures that are coincident with the different types of submovements with a view to providing further clarity or insights around this issue.

## General methods

The following general methods pertain to how the data were handled for all three experiments. Any differences in the protocols between the experiments are indicated within a separate method for each individual experiment.

### Data processing

The aiming movements were captured by an external motion capture system sampling at 200 Hz, which generated three-dimensional position time-series data. These data were smoothed using a 2nd-order, dual-pass Butterworth filter with a low-pass cut-off frequency of 10 Hz. The three-point central difference method was used to calculate the first, second and third derivatives of displacement to obtain velocity, acceleration and jerk, respectively. Movement onset and offset were, respectively, defined by the first moment that velocity reached > 10 mm/s, and returned to < 10 mm/s whilst being > − 10 mm/s, for a period of at least 40 ms (equivalent of 8 samples) (for similar procedures, see Meyer et al. 1988; Chua and Elliott 1993; Teasdale et al. 1993; Sainburg et al. 2003; Elliott and Hansen 2010; Rand and Stelmach 2011).

**Fig. 1:**
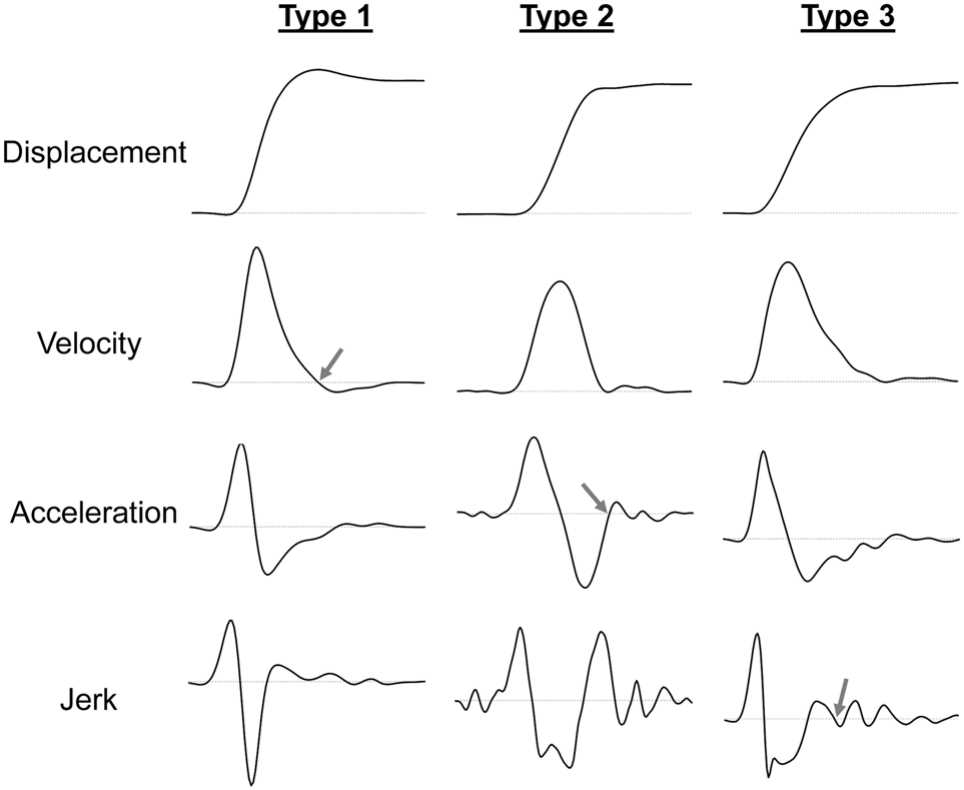
Participant sample profiles of displacement, velocity, acceleration and jerk that are related to different types of submovement. The moment of each submovement is indicated by dark grey arrows including positive-to-negative zero-line crossing in velocity (type 1), negative-to-positive zero-line crossing in acceleration (type 2) and positive-to-negative zero-line crossing in jerk (type 3)

A search for submovements within the primary movement axis was conducted by traversing forward sample-by-sample from the moment of peak negative acceleration (Fig. 1). That is, we identified any moments where there was a positive-to-negative zero-line crossing in velocity (type 1) (associated with a reversal following an overshoot), negative-to-positive zero-line crossing in acceleration (type 2) (associated with re-acceleration following an undershoot), and/or positive-to-negative zero-line crossing in jerk (type 3) (associated with discontinuities/braking following an undershoot).^1^ If at least one of these criteria was fulfilled, then it would have to be maintained for a period of at least 40 ms in order for it to be registered as a submovement. On the rare occasion that there was a combination of these criteria being met, then the first one to be identified was taken as the beginning of a secondary submovement (Dounskaia et al. 2005, 2009; Khan et al. 2006; cf. Elliott et al. 2014). In the event of detecting a type 1 submovement, then we identified the subsequent negative velocity peak with a view to further delineating the functional basis of this submovement phase. Presumably, a more definitive attempt to reverse the movement and reach closer to the target would coincide with a higher magnitude corrective impulse.

### Dependent measures and data analysis

Prior to any statistical analysis, we first removed any trials that landed in excess of 50 mm away from the target because they could be regarded as mere artefacts of failed data processing. Of interest, none of the upcoming analyses replicated any of the previous studies from which these data originated. For the first set of analyses, we compared the proportion of submovement types, including type 1, type, 2, type 3 and none (single component), using a one-way repeated-measures ANOVA. In order to ensure there was a limited trade-off between speed and accuracy, we compared submovement types, including type 1, type 2 + 3 and none,^2^ using a one-way repeated-measures ANOVA for radial error (RE) (distance between the target-centre and terminal endpoint taken with respect to the movement offset velocity threshold, i.e. √x^2^ + y^2^) and movement time (difference in time between the movement onset and offset velocity thresholds).

Therein, we further decomposed type 1 submovements into functional and non-functional by effectively assessing whether the secondary submovement was corrective in nature using the index of error correction effectiveness (IECE): RE_primary_–RE_secondary_/RE_primary_ + RE_secondary_ (see Khan and Franks 2003; Khan et al. 2006).^3^ When IECE was positive (> 0), we considered a trial to be functional, and when it was negative (≤ 0), we considered it to be non-functional (e.g. Robinson et al. 2014).

At this juncture, we compared the proportion of functional and non-functional type 1 submovements using a paired-samples *t*-test. In order to corroborate this categorisation and observe the nature of any corrections that had unfolded, we compared functional type 1, non-functional type 1 and type 2 + 3 submovements using a one-way repeated-measures ANOVA for the displacement of the primary and secondary submovements. Further still, we compared functional and non-functional type 1 submovements for the subsequent negative velocity peak using a paired-samples t-test (N.B., next to no possibility for a negative velocity peak within type 2 + 3 submovements).

To examine the potential of type 1 submovements being generally coincident with more rapid movements, and type 2 and/or type 3 submovements being coincident with slower movements (Fradet et al. 2008a; Hsieh et al. 2017, 2019, 2022), we compared each of these types of submovement using a one-way repeated-measures ANOVA for measures of movement time, time to the end of the primary submovement, time spent completing the secondary submovement, and magnitude of the initial velocity peak.

For comparisons involving ANOVA, the Sphericity assumption was checked using Mauchly’s test (*p* < 0.05). In the event of a violation, then the Huynh–Feldt correction was adopted when ɛ was ≥ 0.75, whereas the Greenhouse–Geisser correction was adopted when ɛ was < 0.75 (original Sphericity-assumed degrees-of-freedom are reported). Any significant effects were further decomposed using the Holm–Bonferroni post hoc procedure (for the sake of brevity, we report only on pairwise comparisons where there was a significant difference). Meanwhile, the effect size measure of interest was partial eta-squared (*ƞ*_*p*_^*2*^). For comparisons involving a t-test, the normality assumption was checked using a combination of the Shapiro–Wilk test (*p* < 0.05) and experimenter observation of the frequency distribution profile. Meanwhile, the effect size measure of interest was Cohen’s *d*. For each of the statistical tests alpha was set at *p* < 0.05.

## Experiment 1

### Method

#### Participants

This study featured a total of 9 participants (age range = 21–40 years; 7 males, 2 females). Participants were self-reported right-hand dominant with normal or corrected-to-normal vision, and no known neurodiverse condition. Since there was 1 participant that failed to register a non-functional type 1 submovement, and another participant that failed to register a single component movement (*n* = 2), they were removed prior to any analyses involving these particular levels of the within-subject submovement variable.

#### Apparatus and task

For full details, see the Method section within Roberts et al. (2022). Participants sat in front of an LCD monitor (47.5 × 27.0 cm; temporal resolution = 60 Hz, spatial resolution = 1280 × 800 pixels) and graphics digitiser (GTCO Calcomp Drawing Board VI), which were each connected to an adjacent computer that controlled the experiment using Matlab (2018b, The Mathworks Inc., Natick, MA) running Psychtoolbox (version 3.0.11).

Participants were tasked with a single or discrete, three-dimensional aiming movement in a predominantly mediolateral axis (left-to-right) as quickly and accurately as possible with the dominant right upper limb using a stylus pen. The aiming movement was executed over a piece of paper secured to the surface of the digitiser by placing it on the underside of a transparent acrylic sheet that was attached near the top. The paper featured a printed home position and target. The home position was represented by a cross-hair (10 × 2-mm lines), whilst the target was either a cross-hair or solid filled circle that varied in size and assumed a set amplitude of 243 mm (centre-to-centre) (see later within *Procedures*). A retro-reflective marker was attached near the tip of the stylus and detected by a Vicon Vantage camera system (Vicon Motion Systems Ltd, Oxford, UK) with the sampling frequency set to 200 Hz.

### Procedures

Participants would signal their readiness by pressing down on the tip of the stylus over the home position. Following a 2-s delay, a 100-ms tone (750 Hz) would sound to signal the participant to move towards the target. In the original study, participants attempted this aiming task over two separate sessions including a temporally constrained aim towards a cross-hair (Session 1—Baseline Trials), and an accuracy-constrained aim featuring a circle (Session 2—Accuracy-Constrained Trials). The first session was intended to capture the participant’s own inherent variability for a typical target-directed aiming movement lasting 400–500 ms. Here, participants had to reach as close as possible towards the centre of a cross-hair target, but within the fore mentioned criterion time. Participants continued this session until they reached within this time window for 30 trials, which was facilitated by augmented feedback of the precise movement times displayed on the adjacent monitor immediately following each attempt. Therein, the second session featured circular targets, where participants had to try to reach inside the target, but as quickly as possible and no longer received augmented feedback on their movement time. The size of the forementioned targets was scaled in accordance to the previously captured variability so that they could be equated to 38.30%, 68.26%, 86.64% and 95.44% of the spatial distribution of individual participant primary movements. Each target was presented within a block of 30 trials, whilst the order of the blocks was pseudo-randomised using a Latin-Square design. For the purposes of the present analysis, we focussed solely on the third possible target equating to 86.64% of the spatial distribution (× 3 within-participant SD; ± 1.5), because it generally manifested in the most overshooting (see Fig. 2A from Roberts et al. 2022). The target sizes ranged between 6.29 and 14.35 mm across participants.

**Fig. 2:**
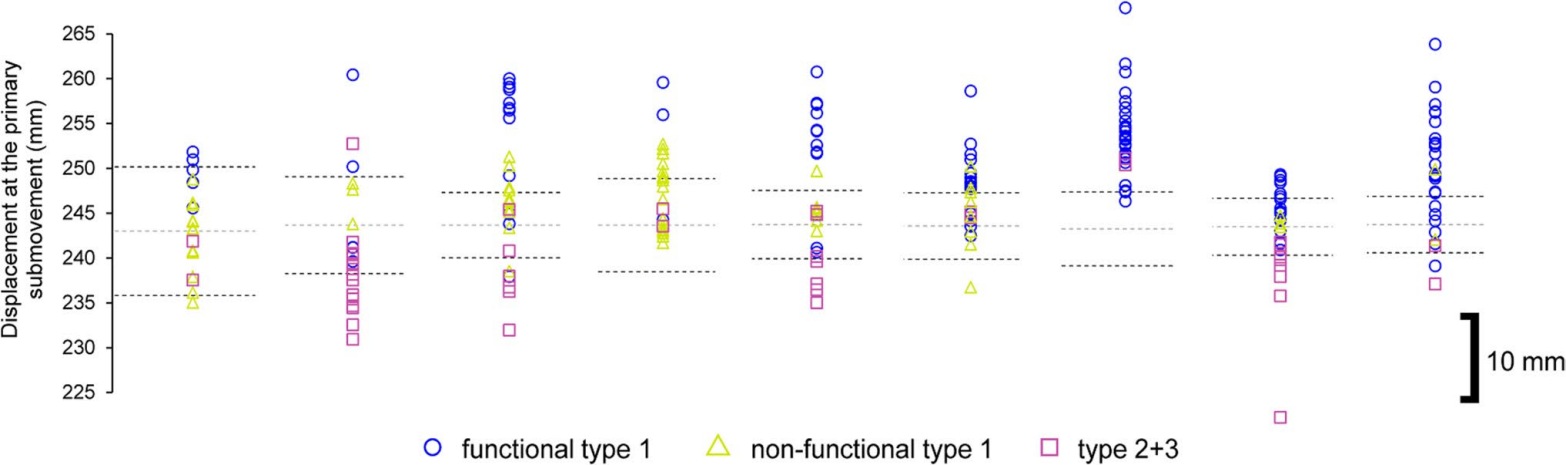
Individual participant displacement of the primary submovement as a function of submovement type (see legend) across all trials. Target-centre is represented by a light grey dotted line, whilst the upper and lower target boundaries are represented by thin black dotted lines (N.B., 10-mm scale inset). Of interest, the target boundaries across participants reflect the subtle variation in designated targets as a result of the within-participant scaling of target size

### Results

#### Proportion of submovement types

The proportion of submovement types revealed a significant main effect of type, *F*(3,24) = 35.45, *p* < 0.001, *ƞ*_*p*_^*2*^ = 0.82, with a significantly larger number of trials featuring type 1 compared to type 2 (*p* < 0.001), type 3 (*p* < 0.001) and none (*p* < 0.001) (see Table 1).

**Table 1:**
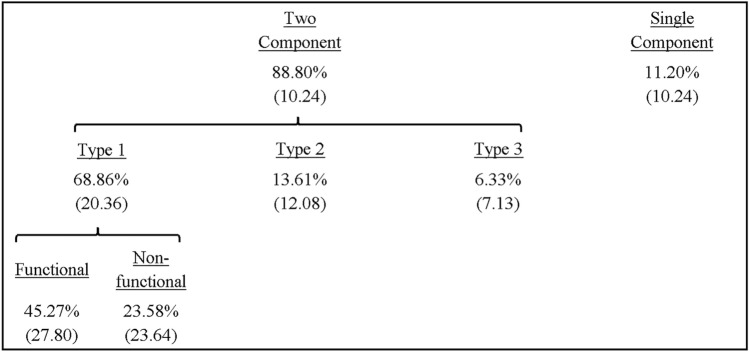
Mean (± SD) percentage of submovement types across all participants including type 1 (functional, non-functional), type 2, type 3 and none

For RE, there was no significant main effect of type, *F*(2,14) = 2.98, *p* = 0.08, *ƞ*_*p*_^*2*^ = 0.30 (*grand M* = 5.39 mm, *SE* = 0.87). Meanwhile, the movement time showed a significant main effect of type, *F*(2,14) = 60.02, *p* < 0.001, *ƞ*_*p*_^*2*^ = 0.90, with a significantly shorter time for movements with none (*M* = 404.02 ms, *SE* = 20.25) compared to type 1 (*M* = 537.40 ms, *SE* = 21.46) (*p* < 0.001) and type 2 + 3 (*M* = 546.85 ms, *SE* = 23.94) (*p* < 0.001).

#### Function and characteristics of type 1 submovements

The individual participant distribution of submovement types and their displacement can be observed in Fig. 2. Most of the trials featuring a type 1 submovement were categorised as functional, although there was no significant difference between the proportion of functional and non-functional type 1 submovements, *t*(8) = 1.36, *p* = 0.21, *d* = 0.45.

**Fig. 3:**
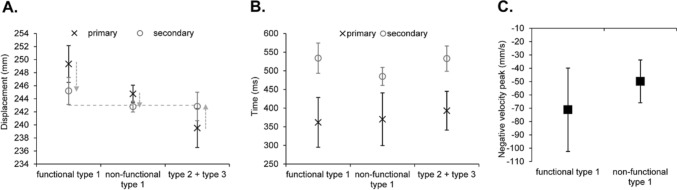
Mean displacement (**A**) (light grey dotted line represents target-centre, and light grey arrows indicate the direction from the primary to secondary submovements) (N.B., no target boundaries were specified due to them being participant-specific) and time (**B**) of the primary (x in black) and secondary (o in grey) submovements, and magnitude of the negative velocity peak following the primary submovement (**C**) as a function of types of submovement. Error bars represent the between-participant standard deviation

For the displacement of the primary submovement, there was a significant main effect of type, *F*(2,14) = 42.84, *p* < 0.001, *ƞ*_*p*_^*2*^ = 0.86, with a significantly longer displacement for movements containing a functional compared to non-functional type 1 (*p* = 0.002), which was significantly longer still compared to the type 2 + 3 (*p* = 0.002) (see Fig. 3a). The displacement of the secondary submovement showed a significant main effect of type, *F*(2,14) = 38.60, *p* < 0.001, *ƞ*_*p*_^*2*^ = 0.85, with a significantly longer reversal for the functional compared to non-functional type 1 (*p* = 0.016), which was significantly longer still compared to the type 2 + 3 (*p* < 0.001). Meanwhile, the magnitude of the negative velocity peak showed a significantly greater magnitude for the functional compared to non-functional type 1, *t*(8) = 2.91, *p* = 0.023, *d* = 1.03 (see Fig. 3c).

For movement time, there was a significant main effect of type, *F*(2,14) = 8.42, *p* = 0.004, *ƞ*_*p*_^*2*^ = 0.55, with a significantly shorter time for movements containing a non-functional type 1 compared to both the functional type 1 (*p* = 0.011) and type 2 + 3 (*p* = 0.013) (see Fig. 3b). Further inspection of the time including the time to the primary submovement showed a main effect of type that approached conventional levels of significance, *F*(2,14) = 3.65, *p* = 0.053, *ƞ*_*p*_^*2*^ = 0.34, where there appeared to be shorter times for movements with a functional and non-functional type 1 compared to type 2 + 3. For time within the secondary submovement, there was a significant main effect of type, *F*(2,14) = 9.54, *p* = 0.002, *ƞ*_*p*_^*2*^ = 0.58, with a significantly shorter time for the non-functional compared to functional type 1 (*p* = 0.002). For the magnitude of the initial velocity peak, the main effect of type approached conventional levels of significance, *F*(2,14) = 3.23, *p* = 0.07, *ƞ*_*p*_^*2*^ = 0.32, with a greater magnitude for movements with a functional (*M* = 1407.44 mm/s, *SE* = 104.76) and non-functional (*M* = 1393.46 mm/s, *SE* = 112.52) type 1 compared to type 2 + 3 (*M* = 1316.46 mm/s, *SE* = 84.56).

## Experiment 2

### Method

#### Participants

This study featured a total of 14 participants (age range = 18–30 years), although only 11 participants qualified for the analysis due to them being susceptible to the socio-comparative threat that was designed to induce stress (see later within *Procedures*). Participants were self-reported right-hand dominant with normal or corrected-to-normal vision, and no known neurodiverse condition. Because there was 1 participant that failed to register a non-functional type 1 submovement, and another participant that failed to register both a type 2 + 3 submovement and single component movement (*n* = 2), they were removed prior to any analyses involving these particular levels of the within-subject submovement variable.

#### Apparatus and task

For full details, see the Method section within Roberts et al. (2018). Participants stood over an LCD monitor (temporal resolution = 60 Hz, spatial resolution = 1024 × 768 pixels), which was connected to an adjacent computer that controlled the experiment using E-prime (Psychology Software Tools Inc., Sharpsburg, PA). The monitor was covered by a 5-mm thick transparent Plexiglas and oriented horizontally within a wooden frame that was mounted on top of a steel ledge. The height of the ledge was adjusted along a vertical stand so the monitor was approximately aligned with the participant’s hip joint.

Participants were tasked with a single or discrete, three-dimensional aiming movement in a predominantly posteroanterior axis (back-to-front) as quickly and accurately as possible using the index finger of their dominant right upper limb. The aiming movement was executed over the monitor and attached Plexiglas surface with a circular home position (10-mm width) and target (10-mm width) being displayed at a set amplitude of 240 mm (centre-to-centre). An infra-red marker was attached to the tip of the right index finger, which was captured by an Optotrak 3020 camera system (Norther Digital Instruments, Waterloo, ON).

### Procedures

Participants would start each trial by placing their right index finger over the home position. Following a random 800–2800-ms foreperiod, the target would appear in the distance along the participant midline to signal the participant to move. In the original study, participants first completed a familiarisation of the task. Therein, they aimed under instructions that were intended to separately elicit either low or high performance-related stress. That is, the low stress block simply involved aiming as quickly and accurately as possible with no further consequence or assessment of performance. The high stress block additionally featured a socio-comparative threat, where participants were informed that their current speed and accuracy performance rendered them one of the worst performers by being in the bottom third of overall participant rankings. There were 30 trials within each block with the order of the stress conditions being counter-balanced between participants. If the low stress block featured first, then participants simply continued with the protocol following familiarisation without any indication of being recorded. Alternatively, if it featured second following high stress, then participants were led to believe that it was of no consequence because it merely acted as the penultimate block. If the high stress block featured first, then the false performance feedback was associated with the previous familiarisation. Alternatively, if it featured second following low stress, then the false performance feedback was associated with the previous low stress block. For the purposes of the present study, we isolated our analysis to the high stress condition, where there was a greater incidence of overshooting.

### Results

#### Proportion of submovement types

The proportion of submovement types revealed a significant main effect of type, *F*(3,30) = 16.37, *p* = 0.001, *ƞ*_*p*_^*2*^ = 0.62, with a significantly larger number of trials featuring type 1 compared to type 2 (*p* < 0.001) and type 3 (*p* = 0.001), whilst there was a significantly larger number for none compared to type 2 (*p* = . 011) (see Table 2).

**Table 2:**
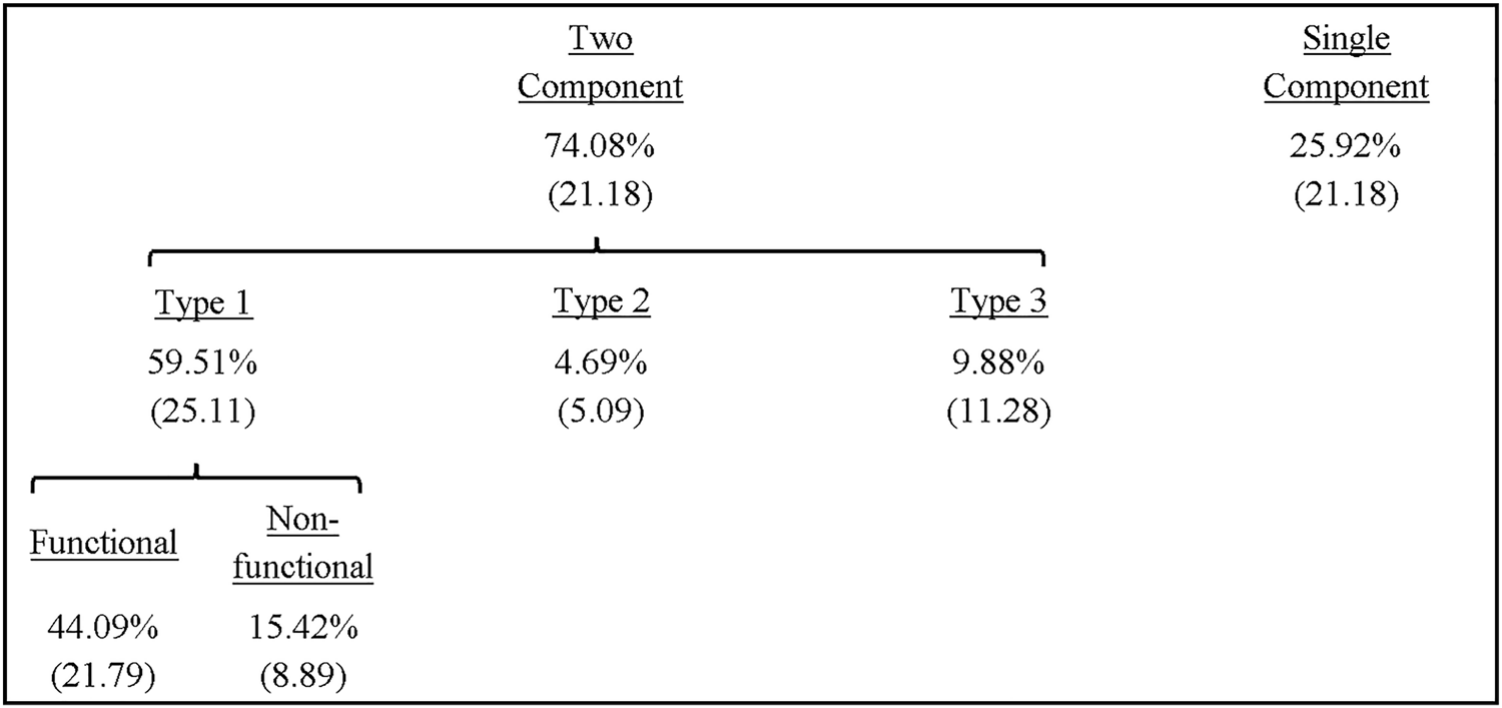
Mean (± SD) percentage of submovement types across all participants including type 1 (functional, non-functional), type 2, type 3 and none

For RE, there was no significant effect of type, *F*(2,18) = 0.85, *p* = 0.45, *ƞ*_*p*_^*2*^ = 0.09 (*grand M* = 5.80 mm, *SE* = 0.51). Meanwhile, the movement time showed a significant main effect of type, *F*(2,18) = 19.60, *p* < 0.001, *ƞ*_*p*_^*2*^ = 0.69, with a significantly shorter time for movements with none (*M* = 374.80 ms, *SE* = 16.71) compared to type 1 (*M* = 449.89 ms, *SE* = 16.16) (*p* < 0.001) and type 2 + 3 (*M* = 475.95 ms, *SE* = 19.42) (*p* < 0.001).

#### Function and characteristics of type 1 submovements

**Fig. 4:**
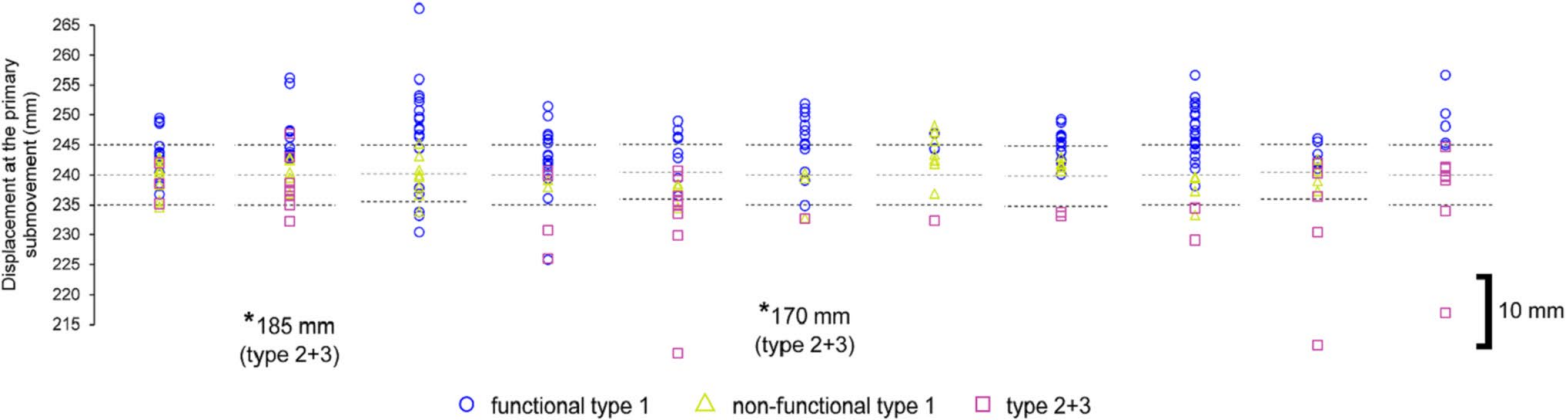
Individual participant displacement of the primary submovement as a function of submovement type (see legend) across all trials. Target-centre is represented by a light grey dotted line, whilst the upper and lower target boundaries are represented by thin black dotted lines (N.B., 10-mm scale inset)

The individual participant distribution of submovement types and their displacement can be observed in Fig. 4. There was a significantly higher proportion of functional compared to non-functional type 1 submovements, *t*(10) = 4.35, *p* = 0.001, *d* = 1.31.

**Fig. 5:**
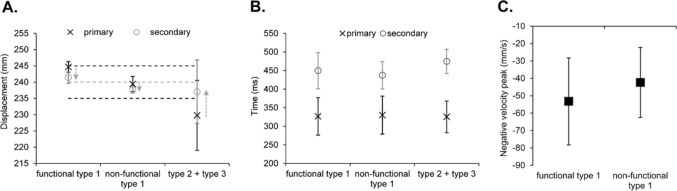
Mean displacement (**A**) (light grey dotted line represents target-centre, black dotted lines represent the upper and lower target boundaries, and light grey arrows indicate the direction from the primary to secondary submovements) and time (**B**) of the primary (x in black) and secondary (o in grey) submovements, and magnitude of the negative velocity peak following the primary submovement (**C**) as a function of types of submovement. Error bars represent the between-participant standard deviation

For the displacement of the primary submovement, there was a significant main effect of type, *F*(2,16) = 12.84, *p* = 0.005, *ƞ*_*p*_^*2*^ = 0.62, with a significantly longer displacement for movements containing a functional compared to non-functional type 1 (*p* < 0.001), which was significantly longer still compared to the type 2 + 3 (*p* = 0.022) (see Fig. 5a). The displacement of the secondary submovement showed a significant main effect of type, *F*(2,16) = 9.61, *p* = 0.013, *ƞ*_*p*_^*2*^ = 0.55, with a significantly longer reversal for the functional compared to non-functional type 1 (*p* = 0.03), which was significantly longer still compared to the type 2 + 3 (*p* = 0.022). Meanwhile, the magnitude of the negative velocity peak showed a significantly greater magnitude for the functional compared to non-functional type 1, *t*(10) = 2.41, *p* = 0.039, *d* = 0.76 (see Fig. 5c).

For movement time, there was no significant main effect of type, *F*(2,16) = 2.67, *p* = 0.13, *ƞ*_*p*_^*2*^ = 0.23 (see Fig. 5b). Likewise, there was no significant main effect of type for the time to the primary, *F*(2,16) = 0.17, *p* = 0.73, *ƞ*_*p*_^*2*^ = 0.02, nor within the secondary, *F*(2,16) = 2.67, *p* = 0.10, *ƞ*_*p*_^*2*^ = 0.25, submovements. For the magnitude of the initial velocity peak, the main effect of type approached conventional levels of significance, *F*(2,16) = 3.17, *p* = 0.07, *ƞ*_*p*_^*2*^ = 0.28 (*grand M* = 1412.34 mm/s, *SE* = 88.82), with a greater magnitude for movements with a functional (*M* = 1452.38 mm/s, *SE* = 101.47) and non-functional (*M* = 1425.18 mm/s, *SE* = 102.65) type 1 compared to type 2 + 3 (*M* = 1359.45, *SE* = 65.39).

## Experiment 3

### Method

#### Participants

This study featured a total of 16 participants (age range = 22–38 years; 11 males, 5 females). Participants were self-reported right-hand dominant with normal or corrected-to-normal vision, and no known neurodiverse condition. Since there were 3 participants that failed to register a type 2 + 3 submovement, 2 participants failing for a single component movement, 1 participant failing for both a type 2 + 3 submovement and single component movement, and 1 participant failing for a non-functional type 1 submovement (*n* = 7), they were removed prior to any analyses involving these particular levels of the within-subject submovement variable.

#### Apparatus and task

For full details, see the Method section within Roberts et al. (2016). Participants stood over an LCD monitor (temporal resolution = 60 Hz, spatial resolution = 1024 × 768 pixels), which was connected to an adjacent computer that controlled the experiment using E-prime (Psychology Software Tools Inc., Sharpsburg, PA). The monitor was covered by a 5-mm thick transparent Plexiglas and oriented horizontally within a wooden frame that was mounted on top of a steel ledge. The height of the ledge was adjusted along a vertical stand so the monitor was approximately aligned with the participant’s hip joint.

Participants were tasked with a single/discrete, three-dimensional aiming movement in a predominantly mediolateral axis (left-to-right) as quickly and accurately as possible using the index finger of their dominant right upper limb. In a similar vein to Experiment 2, the aiming movement was executed over the monitor and attached Plexiglas surface with five outlined square placeholders (1-mm thickness; 10 × 10-mm squares). These placeholders were equidistant to each other with a 5-mm spacing and set at the following amplitudes: 182, 199, 216, 233, and 250 mm (centre-to-centre) (assuming indices of difficulty of 5.19, 5.31, 5.43, 5.54 and 5.64 bits, respectively). An infra-red marker was attached to the tip of the right index finger, which was captured by an Optotrak 3020 camera system (Norther Digital Instruments, Waterloo, ON).

### Procedures

Participants started each trial by placing their right index finger over the home position. Following a random 800–2800-ms foreperiod, a target would be highlighted by one of the five possible placeholders becoming filled to signal the participant to move towards that location. In the original study, one of the five possible targets could be selected at random with the exception that every possible target would be selected once in every 5 trials. There were 20 trials per target, which equated to a total of 100 trials. For the purposes of the present analysis, we considered only the last placeholder owing to it manifesting in more overshooting, which incidentally coincides with what has been known as a violation in Fitts’ Law (i.e. shorter than expected movement time; e.g. Adam et al. 2006; Malone et al. 2023).

### Results

#### Proportion of submovement types

The proportion of submovement types revealed there was a significant main effect of type, *F*(3,42) = 89.78, *p* < 0.001, *ƞ*_*p*_^*2*^ = 0.86, with a significantly larger number of trials featuring type 1 compared to type 2 (*p* < 0.001), type 3 (*p* < 0.001) and none (*p* < 0.001) (see Table 3).

**Table 3:**
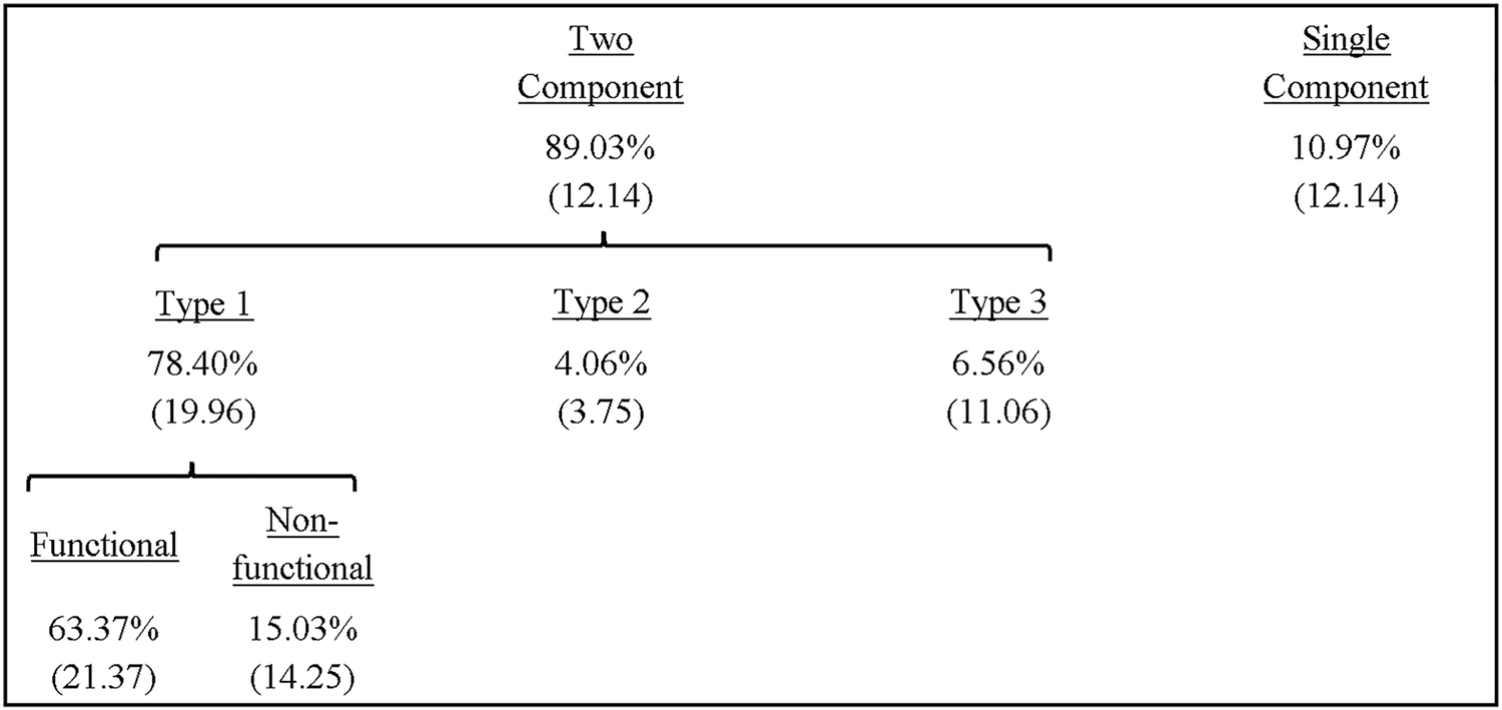
Mean (± SD) percentage of submovement types across all participants including type 1 (functional, non-functional), type 2, type 3 and none

For RE, there was no significant main effect of type, *F*(2,18) = 1.00, *p* = 0.39, *ƞ*_*p*_^*2*^ = 0.10 (*grand M* = 5.50 mm, *SE* = 0.75). Meanwhile, the movement time showed a significant main effect of type, *F*(2,18) = 33.47, *p* < 0.001, *ƞ*_*p*_^*2*^ = 0.79, with a significantly shorter time for movements with none (*M* = 346.30 ms, *SE* = 15.61) compared to type 1 (*M* = 453.81 ms, *SE* = 19.49) (*p* < 0.001) and type 2 + 3 (*M* = 468.67 ms, *SE* = 13.34) (*p* < 0.001).

#### Function and characteristics of type 1 submovements

**Fig. 6:**
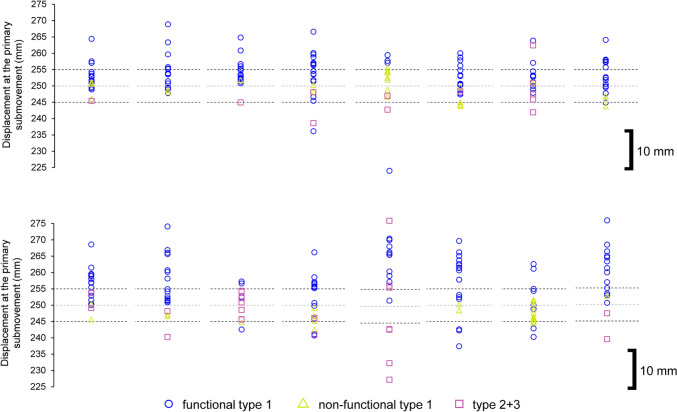
Individual participant displacement of the primary submovement as a function of submovement type (see legend) across all trials. Target-centre is represented by a light grey dotted line, whilst the upper and lower target boundaries are represented by thin black dotted lines (N.B., 10-mm scale inset)

The individual participant distribution of submovement types and their displacement can be observed in Fig. 6. There was a significantly higher proportion of functional compared to non-functional type 1 submovements, *t*(15) = 6.37, *p* < 0.001, *d* = 1.59.

**Fig. 7:**
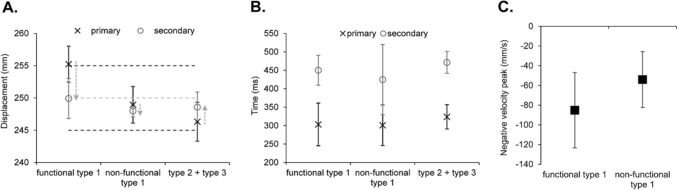
Mean displacement (**A**) (light grey dotted line represents target-centre, black dotted lines represents the upper and lower target boundaries, and light grey arrows indicate the direction from the primary to secondary submovements) and time (**B**) of the primary (x in black) and secondary (o in grey) submovements, and magnitude of the negative velocity peak following the primary submovement (**C**) as a function of types of submovement. Error bars represent the between-participant standard deviation

For the displacement of the primary submovement, there was a significant main effect of type, *F*(2,20) = 26.37, *p* < 0.001, *ƞ*_*p*_^*2*^ = 0.73, with a significantly longer displacement for movements containing a functional type 1 compared to non-functional type 1 (*p* < 0.001) and type 2 + 3 (*p* < 0.001), whilst there was no significant difference between the non-functional type 1 and type 2 + 3 (*p* = 0.10) (see Fig. 7a). The displacement of the secondary submovement showed a significant main effect of type, *F*(2,20) = 31.83, *p* < 0.001, *ƞ*_*p*_^*2*^ = 0.76, with a significantly longer reversal for the functional compared to non-functional type 1 (*p* < 0.001), which was significantly longer still compared to the type 2 + 3 (*p* = 0.003). Meanwhile, the magnitude of the negative velocity peak showed a significantly greater magnitude for the functional compared to non-functional type 1, *t*(14) = 4.64, *p* < 0.001, *d* = 1.20 (see Fig. 7c).

For movement time, there was no significant main effect of type, *F*(2,20) = 1.71, *p* = 0.21, *ƞ*_*p*_^*2*^ = 0.15 (see Fig. 7b). However, the time of the primary submovement showed a significant main effect of type, *F*(2,20) = 4.37, *p* = 0.049, *ƞ*_*p*_^*2*^ = 0.30, with a shorter time for movements with a functional (*p* = 0.075) and non-functional (*p* = 0.037) type 1 compared to type 2 + 3, although these pairwise comparisons failed to reach significance. Meanwhile, the time within the secondary submovement showed there was no significant main effect of type, *F*(2,20) = 0.67, *p* = 0.47, *ƞ*_*p*_^*2*^ = 0.06. Finally, the magnitude of the initial velocity peak showed there was a significant main effect of type, *F*(2,20) = 8.43, *p* = 0.002, *ƞ*_*p*_^*2*^ = 0.46, with a greater magnitude for movements with a functional (*M* = 1629.86 mm/s, *SE* = 79.49) (*p* = 0.01) and non-functional (*M* = 1655.31 mm/s, *SE* = 97.44) (*p* = 0.008) type 1 compared to type 2 + 3 (*M* = 1487.23 mm/s, *SE* = 55.02).

## Discussion

The present study revisited previous datasets featuring a higher than typical incidence of type 1 submovements (Roberts et al. 2016, 2018, 2022). The classic two-component interpretation of speed-accuracy in manual aiming would have it that these submovements are a manifestation of a correction following an initial error within the limb trajectory (Elliott et al. 2001, 2010, 2017; Woodworth 1899). Alternatively, it has been suggested that such submovements are merely a by-product of some entirely separate non-functional mechanical artefact (Dounskaia et al. 2005; Fradet et al. 2008a; see also, Plamondon and Alimi 1997). With this in mind, it was reasoned that by further categorising type 1 submovements as either functional and non-functional, we could more clearly highlight the origin of these particular submovements. To elucidate, we identified the type 1 submovements that reached closer to the target as functional under the assumption that they were likely corrective in nature, whilst those that did not were identified as non-functional because they failed to demonstrate any error-reducing outcome.

We confirmed that most of the trials did indeed consist of type 1 submovements (Experiment 1–3 M range = 60–78% of trials). Of these type 1 submovements, most of them were in fact functional rather than non-functional (Experiment 1–3 M range = 65–81% of type 1 submovement trials). That is, the limb initially overshoots target-centre within the primary submovement before it is reversed in the opposing direction during the secondary submovement. This submovement is typically seen as a correction based on sensory feedback from the movement (Woodworth 1899), which can be linked more specifically to the external visual afferent information about limb and target location (Elliott et al. 2010, 2017). As a result, this correction is more likely subject to a processing time-lag (Grierson and Elliott 2009) and conscious awareness (Cressman et al. 2007) (for an alternative view of interacting early and late corrections, see Grierson and Elliott 2008; Roberts et al. 2017).

At the same time, it is possible this type of submovement unfolds following the accumulation of potential energy that is related to the viscoelastic properties at the antagonist muscle (Savelberg et al. 2002). To elucidate, the prolonged contraction of the agonist that is responsible for the limb initially overshooting the target can subsequently cause elastic potential energy to build up in the opposing antagonist muscle. Therein, it is possible to convert this energy in a spring-like fashion for the rapid and smooth transition into the opposing movement direction, which can place the limb nearer the intended target. Here, the inherent challenges posed by the time and energy-expenditure when overcoming inertia may be minimised by adopting this alternative approach to the target (Adam et al. 1993; Oliveira et al., 2005). In the context of the present study, there was perhaps sufficient reciprocity between the respective agonist and antagonist muscle groups for this exact feature to be exploited despite the task involving an alternative three-dimensional aiming movement to make contact with a surface in the distance without much sliding.

Importantly, this particular account of functional type 1 submovements may not necessarily be feedback-based, but alternatively feedforward in nature courtesy of inherent low-level postural control mechanisms that can help find an equilibrium point. In this instance, the system attempts to calibrate itself based on a set threshold in muscle length-tension courtesy of the extrafusal alpha (Abend et al. 1982; Bizzi et al. 1984) and/or intrafusal gamma (Feldman 1986) motor neurons. Once this threshold is met, then the system automatically exploits the viscoelastic properties at the opposing antagonist muscle by activating it to stop the limb movement within its tracks and reach a final equilibrium point (see also, Grierson et al. 2011). We do not consider this type of Type 1 submovement as an artefact, but rather as a feedforward corrective process associated with the initial planning of the movement.

This is not to say that a mechanical artefacts are not present in the reported dataset. Indeed, there were also a substantive number of non-functional type 1 submovements (Experiment 1–3 M range = 19–35% of type 1 submovements). These particular instances of a type 1 submovement have also been linked to movement termination and stabilisation processes, whereby the so-called submovement is an artefact of the mechanical oscillations that unfold when trying to abruptly stop the limb from any further movement (Dounskaia et al. 2005; Fradet et al. 2008a). This behaviour is reflected within the stereotypical triphasic EMG pattern that emerges for discrete aiming movements. To elucidate, there is an agonist muscle burst that is responsible for the initial accelerative portion of the movement followed by an antagonist muscle burst that is responsible for the penultimate decelerative portion. Therein, agonist muscle activity once again emerges toward the end with a view to effectively “clamping” the limb near the target (Hallett et al. 1975; Hannaford and Stark 1985; Wadman et al. 1979; see also, Savelberg et al. 2002). Since the current study alternatively featured a surface for the limb to make contact and immediately stop, it could be argued that this prohibited the fore mentioned triphasic pattern of muscle activity including the so-called “clamping”. However, the surface of interest was not perpendicular to the primary axis of movement, but instead ran parallel with it. Thus, the accelerative and decelerative portions of the movement were still likely followed by the need to “clamp” the limb.

Alongside this, more mechanical explanation is the expectation that type 1 submovements coincide with faster movements. However, the present findings were somewhat mixed in this regard as non-functional type 1 submovements were associated with shorter movement times in Experiment 1, but no such differences were apparent in Experiment 2 and 3. Of interest, there were some underlying methodological differences between these experiments including Experiment 1 involving a mediolateral aiming movement with a pointed stylus, whilst Experiment 2 and 3 involved pointing the index finger that could effectively cover the entire target area. That said, to more closely reconcile these discrepancies, it may help to consider how type 1 submovements have been associated with both faster and slower movements. Specifically, a shorter movement time may unfold because of the coincidentally higher velocity magnitude of the initial impulse that then requires a greater counter-acting force (Hsieh et al. 2017), although a longer movement time may alternatively unfold because it can take more time to overcome this limb inertia and switch the agonist–antagonist muscle functions (Elliott et al. 2004). When taken together or operating in-tandem, these factors could effectively cause any potential time differences to average or cancel each other out. However, what is perhaps more likely is the fact that the present study featured a rather limited range of movement times (Experiment 1–3 participant *M* range = 331–672 ms) compared to some other studies (e.g. approx. range = 420–2115 ms; Hsieh et al. 2019), which may have precluded any influence of the type of submovements.

Upon reflection, whilst most studies have been somewhat pre-occupied with the type of submovements involving the classic distinction between first (velocity), second (acceleration) and third (jerk) derivatives of displacement (e.g. Chua and Elliott 1993; Dounskaia et al. 2005; Elliott et al. 2004; Fradet et al. 2008a, b; Worringham 1991), the present study indicates how a further assessment surrounding function may be warranted. Indeed, our present categorisation revealed a clearly longer displacement in the primary and secondary (reversal) submovements for functional compared to non-functional type 1 submovements. In addition, there was a higher negative velocity peak within the secondary submovement for functional compared to non-functional type 1 submovements, which could be adapted to further contextualise the origin or nature of type 1 submovements for any future-related studies (e.g. < -50 mm/s for the identification of a corrective reversal). Indeed, it stands to reason that an initially longer primary submovement that more greatly overshoots the target can accommodate a slightly more abrupt and forceful movement in the opposing direction for the secondary submovement to quickly reach nearer the target.

At this juncture, it is relevant to consider the implications for existing models involving submovement structure. Namely, the optimised submovement model predicts primary (sub)movements to reach near target-centre (type 1 ≅ type 2, type 3) with a view to limiting the incidence of target error (Meyer et al. 1988), whilst the minimisation model predicts greater undershooting (type 1 < type 2, type 3) with a view to a correction in the secondary submovement (at least in untrained or non-habituated individuals) (Elliott et al. 2004; see also the multiple process model, Elliott et al. 2010, 2017).^4^ Clearly, the comparatively greater overshooting and related type 1 submovements of the present study cannot be definitively explained by these fore mentioned models. Alternatively, it is suggested that the central tendency and subsequent submovement structure is perhaps more flexible, and subject to the precise sensorimotor context. This context may be broadly judged on the basis of perceived cost and likelihood of errors (Neyedli & Welsh 2013; Trommershäuser et al. 2003a,b; 2005; for an in-depth explanation, see Roberts et al., 2021). For example, it is known that individuals tend to steer their movements more greatly away from an adjacent penalty area and into the target area when there is a perceived high cost (e.g. –500 points) and chance (i.e. inherent variability) of an error. In another context, performers tend to undershoot the target with their initial submovement when aiming downward, presumably because they try to avoid corrections to overshoots that would need to be made against gravity (Bennett et al. 2012, Lyons et al. 2006; see also, Burkitt et al. 2017). With respect to the present study, when it is (mis)perceived that there is a limited cost (e.g. time or energy) and/or chance of an error, then overshooting and the related type 1 submovements may unfold as a result of exploiting the elastic potential energy at the opposing antagonist muscle. That said, future research may more adequately address these suggestions by systematically manipulating the parameters or contexts that can precisely modulate submovement structure.

Upon reflection, it is important to also consider the underlying pitfalls or limitations of the present analysis. Indeed, central to our study is the assumption that functional, as opposed to non-functional, type 1 submovements are able to reach closer to the target compared to the preceding primary submovement. This categorisation was perhaps further substantiated by other differences between these submovements including the magnitude of the negative velocity peak. However, it is possible that a small number of trials featuring a so-called non-functional type 1 submovement may have involved an attempt to reach closer to the target, although failed to unfold as intended. Indeed, whilst updating the limb position can often overcome the initial error imposed by signal-dependent noise (Schmidt et al. 1979; Meyer et al. 1988), it is important to recognise that this process in itself is also subject to noise from multiple sources across the sensorimotor system (Faisal et al. 2008). Likewise, it is also possible that a small number of trials with a functional type 1 submovement may have coincidentally reached closer to the target having manifested from the same movement termination and stabilisation processes previously attributed to the non-functional type 1 submovements. With this in mind, we suggest the current analysis is purely indicative, and sheds further light on pre-existing datasets, which could mostly act as a primer for future research in manual aiming and its related submovement structure.

In summary, we find that some recent studies within our lab have accumulated type 1 submovements that are otherwise associated with a time and energy-consuming correction in the form of a reversal secondary submovement (Roberts et al. 2016, 2018, 2022). Here, we examined their function based on the assumption that they should move the limb closer to the target. Across multiple experiments, we find that the majority of these type 1 submovements were in fact functional, although there were a reasonable portion of non-functional ones as well. We attribute the former to feedforward and/or feedback-based processes designed to bring the limb onto the target. That is, an initial feedforward limb trajectory may overshoot the target with a view to exploiting the viscoelastic properties at the antagonist muscle to reverse the movement in the direction of the target. At the same time, online feedback may help detect an overshoot following the primary submovement, which could then be corrected by reducing the error between the limb and target positions (i.e. limb-target control; Elliott et al. 2010, 2017). Meanwhile, there is also the potential for at least some mechanical artefact, where movement termination and stabilisation processes inadvertently cause the velocity zero-crossing that defines a type 1 submovement. As a result, the present study advocates for the further categorisation of submovements subject to determining their true origin or nature. For further insights, we may adapt this logic to the study of submovements following vision and no vision conditions, where we can more definitely disclose the use of online visual feedback for a correction (e.g. Hsieh et al. 2022). If indeed there is a corrective process underpinning the functional category, then these submovements should present themselves more under vision compared to no vision conditions, although the incidence of non-functional submovements should not necessarily differ.

## Data Availability

Data are available upon request via the corresponding author.

## Footnotes

1 Some studies have alternatively earmarked a submovement following inflection points within negative acceleration whilst reaching a certain magnitude within a temporal window (e.g., 10% of the peak absolute magnitude of acceleration and lasting ≥72 ms; Chua and Elliott, 1993). However, this event incidentally corresponds with the same moment of a positive-to-negative zero-line crossing in jerk (type 3).

2 Because type 2 and 3 submovements both manifest from an undershoot, and there were generally a low proportion of these types of submovement, we combined them for any measures to form a single repeated-measures level of type.

3 The original formulation of error correction effectiveness solely featured error within the primary axis, while referring to initial impulse and error correction phases: AEII – AEEC / AEII + AEEC.

4 It is worth noting that the empirical basis for the optimized submovement model was studies involving a one-dimensional, wrist rotation movement that displaced a cursor on a computer screen (Meyer et al. 1988). Meanwhile, the minimization model was primarily based on studies involving three-dimensional aiming in which the entire upper-limb moved from a home position to a physical target (Elliott et al., 2004). Thus, it could be argued that the relative cost of undershooting or overshooting a target was minimal for the former context, but posed a greater cost within the latter context.
